# World Health Organization's Innovative Direct Disbursement Mechanism for Payment of Grassroots Immunization Personnel and Operations in Nigeria: 2004–2015

**DOI:** 10.1093/infdis/jiv485

**Published:** 2016-04-02

**Authors:** Yared G. Yehualashet, Alieu Wadda, Koffi B. Agblewonu, Theophilus Zhema, Al-asi A. Ibrahim, Alhagie Corr, Jennifer Linkins, Pascal Mkanda, Rui G. Vaz, Peter Nsubuga, Daniel Ashogbon

**Affiliations:** 1 World Health Organization, Country Representative Office; 2 National Primary Health Care Development Agency, Abuja, Nigeria; 3 World Health Organization, Regional Office for Africa, Brazzaville, Congo; 4 Global Public Health Solutions, Atlanta, Georgia; 5 World Health Organization, Headquarters, Geneva, Switzerland

**Keywords:** direct disbursement mechanism, supplementary immunization activities, vaccination personnel payments, mobile payment, WHO Nigeria, polio funds management

## Abstract

***Background.*** Following the 1988 World Health Assembly resolution to eradicate polio, the government of Nigeria, with support from partners, has been implementing several rounds of supplementary immunization activities (SIAs) each year. In addition to the technical requirements, the success of the polio eradication initiative depends on timely provision of adequate financial resources. Disbursement of funds for SIAs and payment of allowances to numerous vaccination personnel at the grassroots level are enormous operational challenges in a country the size of Nigeria. Upon donors' request for a transparent and effective payment mechanism, the World Health Organization (WHO), in consultation with national counterparts, created the innovative direct disbursement mechanism (DDM) in 2004. The objective of the DDM was to timely deploy operational funds at the field level and directly pay vaccination personnel allowances at the grassroots level.

***Methods.*** A detailed operational guideline for funds disbursement was developed in close consultation with central and field stakeholders. Multiyear financial resource requirements and operational budgets for every campaign were produced by an interagency-coordinated finance subcommittee. The WHO engaged a bank and an accounting firm as DDM partners to support disbursement of and accounting for the SIA funds, respectively. The 37 WHO field offices were equipped with electronic financial systems to support the DDM process, and temporary payment sites were set up to facilitate payment to vaccination personnel at the grassroots level. Coordination meetings among DDM partners were held regularly to reconcile financial records and address operational challenges.

***Results.*** Between 2004 and 2014, DDM supported 99 polio and nonpolio vaccination campaigns, disbursing more than $370 million to about 16 million beneficiaries across 280 temporary payment sites. To mitigate security risks and reduce operational costs, the WHO and DDM partners introduced mobile payment to vaccination personnel in May 2015 in compliance with national regulations. A total of 97% of the targeted 1871 beneficiaries in 2 pilot sites were successfully paid through mobile payment, although some challenges remain to be addressed.

***Discussion.*** The DDM has met its objectives with a high rate of financial accountability and transparency, despite persistent operational and security challenges. With support from Nigeria, the Pakistan polio vaccination program successfully adopted the DDM. The DDM continues to play an important role in effective implementation of the polio endgame strategy and the national immunization strategic plan. As part of polio legacy planning, we recommend the DDM as a model for other opportunities that involve the engagement of large field-level teams as new vaccines are introduced in Nigeria and elsewhere.

Nigeria is a federal republic with 36 states and a federal capital territory. There are 774 local government authorities (LGAs) and 9565 wards across the nation. Based on the last census, held in 2006, the country had a projected population of >170 million in 2014, which makes it the most populous in Africa [1, 2].

After the 1988 World Health Assembly resolution to eradicate polio, the government of Nigeria, with support from the World Health Organization (WHO) and partners, has implemented several rounds of immunization campaigns against polio each year [3]. During 2003–2004, Nigeria experienced loss of public confidence in oral poliovirus vaccine (OPV), which ultimately led to the suspension of the supplemental immunization activities (SIAs). Consequently, poliovirus had a resurgence within Nigeria and spread to 20 previously polio-free countries [4]. Since then, several efforts have been made to improve program performance. At present, Nigeria still remains one of the 3 polio-endemic countries in the world, along with Pakistan and Afghanistan. The May 2015 Independent Monitoring Board report lauded Nigeria's recent progress and recommended that the government “build resilience” to stay polio free through 2017 [5].

In addition to technical and logistic requirements, the success of the polio eradication initiative (PEI) depends on timely provision of adequate financial resources to implement planned activities. The funds required for immunization activities in Nigeria are quite substantial, owing to operational complexity; the nature of the oil-driven economy, which makes the cost of goods and services expensive; and the large size of the population [1, 6]. According to the 2012–2016 financial resource requirements, the basic aspects of polio operations in Nigeria cost an average of $240 million per annum [7, 8]. Despite continued increase in the cost of polio eradication, economic models have proved that the return on PEI investment are justified [9]. The Global Polio Eradication Initiative (GPEI) estimated that incremental net benefits of the GPEI between 1988 and 2035 are approximately $40 billion–$50 billion [10].

Disbursement of funds for field activities and payment of vaccination personnel allowances down to the LGA and ward levels (wards are the lowest administrative unit in Nigeria) are enormous operational challenges in a country the size of Nigeria, because SIAs are the most labor intensive of the eradication strategies [11]. In Nigeria, >360 000 and >180 000 personnel are engaged to support 1 round of national and subnational polio vaccination campaigns, respectively. The subnational rounds are conducted in 11 states, which were categorized as high risk for poliovirus transmission, based on epidemiologic risk analysis. These high-risk states are Bauchi, Borno, Jigawa, Kaduna, Kano, Katsina, Kebbi, Niger, Sokoto, Yobe, and Zamfara. For polio alone, on average 2 national and 7 subnational rounds are conducted every year.

Prior to 2004, operational funds to support SIAs in Nigeria were deposited by partners in a central dedicated account managed by the central government agency responsible for disbursement of the funds to the field. This mechanism faced a number of challenges, including underpayment or nonpayment of end beneficiaries, delays in payment and retirement, and inadequacy of documentation. According to a study conducted by the World Bank in 2005, nonpayment of entitlements at the LGA level was an endemic problem in Nigeria [12].

Following an audit's recommendations to address the challenges and inadequacies of the old payment system, major donors requested that a new payment mechanism be set up to continue with their financial contributions. The WHO conducted a risk assessment in 2013 and then established an innovative payment scheme, referred to as a “direct disbursement mechanism” (DDM), in 2014.

The objective of the DDM was to resolve the shortcomings of the old disbursement system and meet donors' expectations. Accordingly, the DDM aimed to avail operational funds in time for implementation of field activities, ensure timely payment of eligible vaccination personnel, and ensure transparent and secured payment process to enhance accountability.

In this article, we describe the process and activities involved in management of the DDM and how it provided an innovative solution for direct payment of activities and vaccination personnel at the grassroots level for successful implementation of SIAs.

## METHODS

We conducted a retrospective review of publications of the global and local PEI partners and working groups, WHO internal documents, and unpublished reports to source data for this article.

### DDM as a Collaborative Process

**Figure 1.**
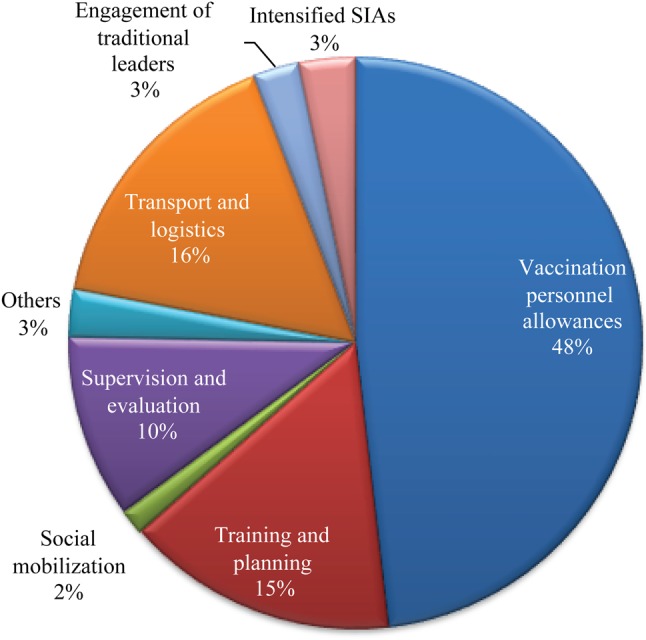
Standard polio campaign operational budget allocations, April 2015. Abbreviation: SIA, supplementary immunization activity.

In line with the spirit of the Paris Declaration on Aid Effectiveness, which called for ownership, harmonization, and mutual accountability, the DDM was designed to function with government and partner involvement [13]. The finance subcommittee of the Interagency Coordination Committee (ICC), chaired by the Nigerian federal government and with the WHO and the United Nations Children's Fund (UNICEF) as members, is responsible for identification of financial resource requirements for PEI activities in Nigeria [7]. For every round of an immunization campaign, the WHO developed a detailed operational budget guided by the financial resource requirements and shared it with the ICC finance subcommittee for review and endorsement. Traditionally, the federal government of Nigeria contributed funds for the procurement of vaccines. Of the total SIAs operational costs, UNICEF disbursed logistic and social mobilization funds, constituting 20%, while the WHO managed the remaining components, which amount to 80% (Figure 1).

**Figure 2.**
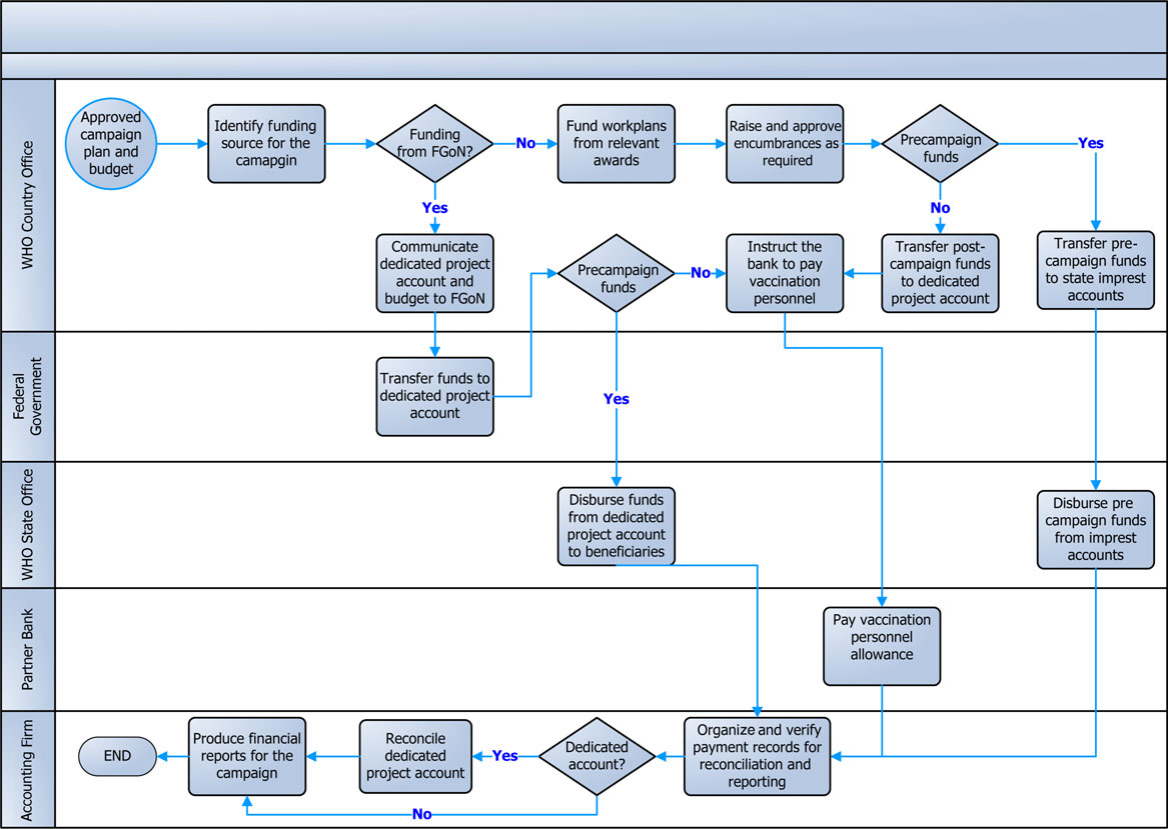
High-level workflow of supplementary immunization activities funds management in Nigeria, May 2015. Abbreviations: FGoN, federal government of Nigeria; WHO, World Health Organization.

To ensure maximum effectiveness and accountability, the WHO engaged the services of a well-established bank and an accounting firm in Nigeria. The bank was responsible for funds disbursement, while the accounting firm provided monitoring, accounting, and reporting services. Figure 2 depicts the high-level work flow of SIA funds in Nigeria.

Broadly, the SIA payments can be divided into 2 categories: payment for campaign activities and payment of allowances for vaccination personnel.

### Payment for Campaign Field Activities

Operational funds were transferred electronically to WHO state offices to implement field-level activities 3 weeks before the beginning of the campaign. The major budget lines under this category included microplanning, training, demand creation supplies, engagement of traditional leaders, monitoring and evaluation, logistics, and social mobilization. The disbursement of these funds was coordinated in consultation with local authorities, as per the state-level operational plans.

To effectively manage the funds, the WHO equipped its central and 37 field offices with an electronic financial system and separate bank accounts. From 2012 onward, the WHO connected each field office with the organization's integrated global management system to process, control and execute management transactions [14]. The global management system is the WHO's enterprise resource planning system that gathers, collates, and produces data, bringing together work flows, procedures, and systems into 1 common system across the WHO in the areas of program planning, human resources, finance, travel, and procurement. The account signatories are profiled in the e-payment platform to fast-track the processing of the financial transactions at the field level. Major suppliers of goods and services are prequalified and profiled in WHO's e-payment platform.

### Payment for Vaccination Personnel

**Figure 3.**
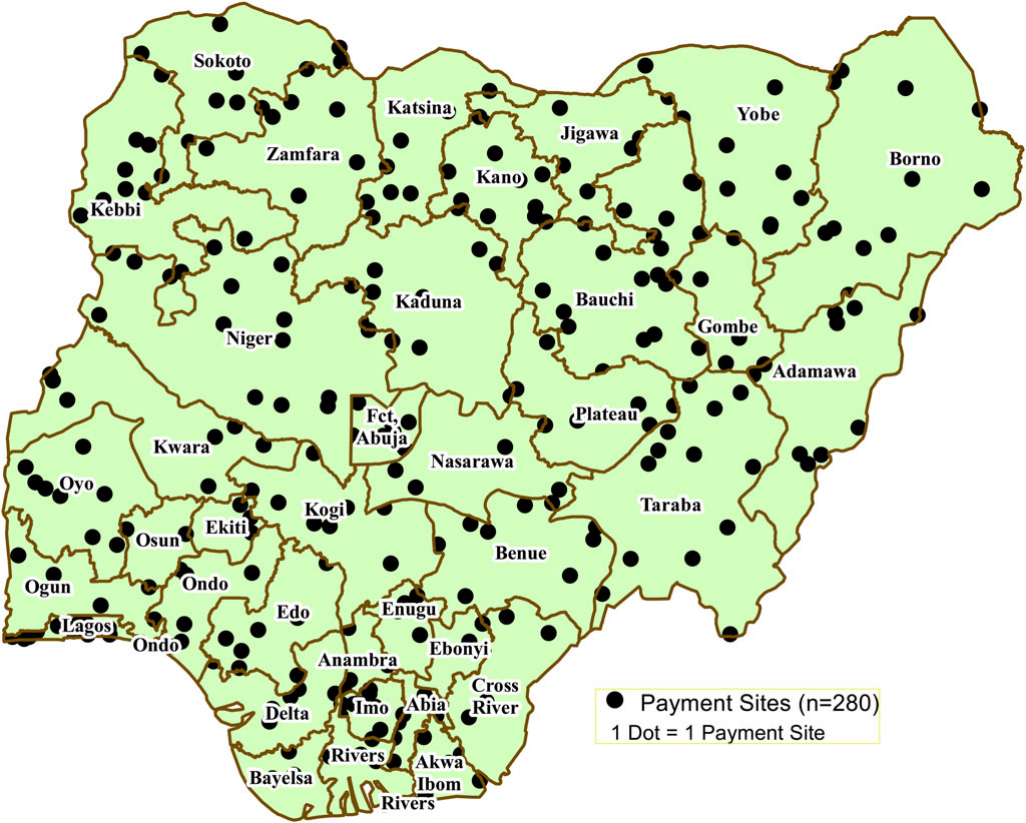
Distribution of current direct disbursement mechanism payment sites (n = 280), May 2015.

Owing to inadequate banking infrastructure at the grassroots level in Nigeria, DDM partners, in collaboration with local authorities, set up about 280 temporary payment sites throughout the country to bring payment facilities closer to the beneficiaries (Figure 3). The bank disbursed allowances to vaccination personnel through its network of local branches or payment agents at designated payment sites.

Allowances for vaccination personnel constituted almost half of the overall operational budget for polio SIAs. In aggregate terms, the cost appears substantial. However, when broken down per beneficiary, as shown in Table 1, it is quite modest. The majority (90%) of the vaccination personnel were paid an average of $5 per day (using the 2014 annual average exchange rate of 164.04 Naira per dollar). The main cost driver for the vaccination personnel allowance component (ie, the number and stipend rates of vaccination teams) was approved by the Nigerian federal government in consultation with implementing partners. The number of vaccination teams was established, in general, on the basis of team workload analysis and other operational and geographical variables.

**Table 1.** Basic Criteria for Determination of Vaccination Personnel Entitlements

Category of Vaccination Personnel	Persons^a^	Proportion of Team Composition, %^b^	Days of Engagement, No.	Daily Stipend Rate, Naira	US$^c^
State team per state	5	0.7	6	1200	7.32
LGA team per LGA	4	1	6	1200	7.32
Ward focal supervisor per ward	1	3	6	1100	6.71
Fixed-post team					
Health worker vaccinator per team	1	5	4	900	7.32
Recorder per team	1	5	4	700	4.27
Special team					
Vaccinator per team	1	5	4	700	4.27
Recorder per team	1	5	4	700	4.27
Town announcer per team	1	5	4	700	4.27
House-to-house team					
Vaccinator per team	1	22	4	700	4.27
Recorder per team	1	9	4	700	4.27
Supervisor per team	1	18	6	1000	6.1
Community leader per team	1	18	4	700	4.27
State technical facilitator per LGA	1^d^	0.3	21	2500	15.24
Independent monitor					
In-process monitors; transport allowance during implementation per LGA	4	2	4	1000	6.1
In-process monitors; stipend per LGA	4		4	1250	7.62
End-process monitors; transport allowance during implementation per LGA	4	1	2	2000	12.19
End-process monitors; stipend per LGA	4		2	2500	15.24

Abbreviation: LGA, local government authority.

^a^ Data are no. of persons per unit, unless otherwise indicated.

^b^ Defined as the share of the level of vaccination personnel category in proportion to the total number of personnel engaged per national polio supplementary immunization activities.

^c^ 2014 annual average exchange rate of N164.04/US$ is used.

^d^ Data are percentage of persons.

Activity cards were distributed to each vaccination campaign worker recruited by local authorities before the commencement of the campaign. A supervisor signed the activity card each day an individual worked on the campaign. The WHO state office compiled the team composition as reported by ward or LGA focal persons. The WHO and the paying bank, with input from state officials and LGA focal persons, jointly developed payment schedules and an operational budget for each site. The WHO's central office reviewed the submissions and issued consolidated payment instructions to the bank. Within 7 days after implementation of the SIAs, the bank commenced payment to each beneficiary upon presentation of the duly signed activity card bearing the picture of the payee. The local authorities physically verified the eligibility of the payees to prevent any irregularity. The WHO administrative focal persons coordinated the whole process and submitted the payment site report to the central office in Abuja.

As new developments emerged in the national financial regulatory landscape, the WHO approached the Central Bank of Nigeria for technical support to explore the possibility of introducing mobile payment. The Central Bank of Nigeria demonstrated keen interest because it saw such projects as an opportunity to drive development and modernization of the payment system, in line with Nigeria's vision of being among the top 20 economies in the world by 2020 [15]. Mobile money is a technology-assisted financial service delivered to the beneficiaries via mobile phone device [16]. A study on the adoption of mobile money in Nigeria revealed that culture is the most important factor influencing the adoption behavior of users of mobile banking in Nigeria [17]. In May 2015, mobile money was piloted to facilitate payment to vaccination personnel in 2 LGAs of Kaduna and Kano states after training the target groups. The particulars of the beneficiaries, including their mobile numbers, were entered into the database. The bank mapped the beneficiaries against local agents to cash out their entitlements upon presentation of balances in their mobile wallet. The current cash-based payment scheme was maintained for individuals without mobile phones.

### Reconciliation and Reporting

The bank compiled the financial documents from all LGAs and aggregated them at the state level. The accounting firm verified the payment/activity cards and other financial documents collected from the bank and the WHO and produced financial and management reports for each SIA.

The WHO regularly convened the meeting of central DDM partners, comprising the bank, the accounting firm, and the company in charge of physical sorting and electronic archiving of the payment cards. This meeting evaluated the DDM's performance during the previous round, assessed the state of partners' preparedness for the next round, addressed any operational challenges, and discussed opportunities for system improvements.

## RESULTS

### Paying the Right Amount to the Right Beneficiary Supporting Polio and Other SIAs

**Figure 4.**
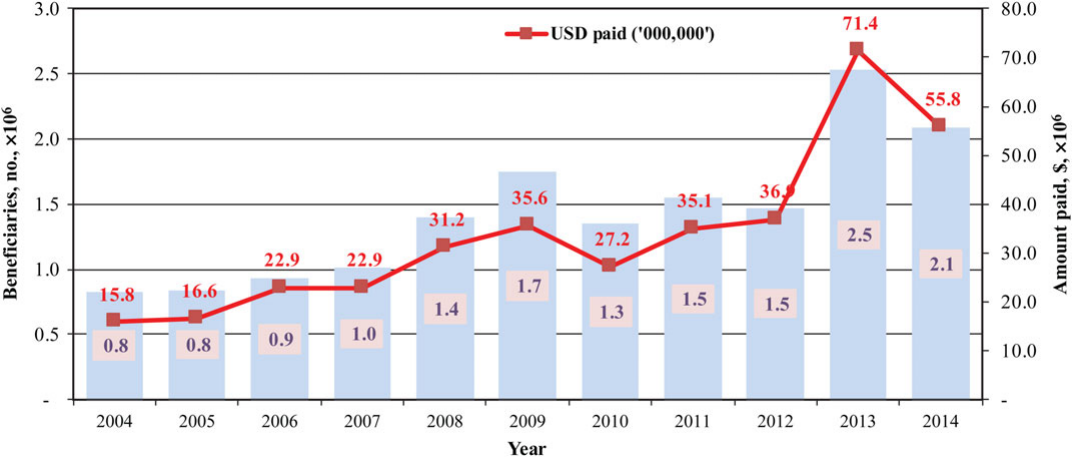
Number of vaccination personnel paid (blue bars and numerals) and amount paid out (red line and numerals) through the direct disbursement mechanism between 2004 and 2014 for various supplementary immunization activities (SIAs). Data are from Akintola Williams Deloitte financial reports on SIAs prepared for World Health Organization Nigeria.

Between 2004 and 2014, 15.7 million beneficiaries supporting 99 immunization campaigns were paid more than $370 million (Figure 4). On average, 1.4 million beneficiaries were paid $34 million per annum for supporting 9 campaigns. When the project was started, it was meant to support polio eradication activities only. However, over the years, the project has successfully managed operational funds for several mass immunization campaigns targeting diseases such as measles, meningitis, and yellow fever.

### DDM as a Leverage to Tap Local Funding Opportunities

The immunization program has leveraged the success of the DDM as a comparative advantage by maintaining donors' confidence to channel substantial amount of grants through the mechanism. Local resource mobilization efforts are boosted, including those of the federal government of Nigeria, whose contribution has been increasing substantially from year to year. Between 2007 and 2014, 29 billion Naira ($194 million, based on various exchange rates used upon receipt of the different tranches over the years) was received for various SIAs from the government and effectively managed through the DDM in line with the memoranda of understanding signed between the government and the WHO.

### Enhanced Financial Management Tools for Effective Payment Mechanism

The WHO's physical presence throughout the country plays important role in the DDM's effectiveness. The demands for direct funds disbursement prompted continued upgrading of the internal WHO financial management tools and systems, to ensure that field operational funds are made available in a timely fashion and that cash flow requirements are managed prudently. After setting up e-payment platforms in all field offices, the WHO fully migrated from cash-based payment to direct electronic payment to suppliers of demand creation materials and allowances to state teams, independent monitors, quality surveyors, as well as state technical facilitators involved in every SIAs. This was instrumental in mitigating financial and security risks. Transactions produced at the field-office level are monitored at the national and global levels in real time.

### High Rate of Financial Accountability

**Figure 5.**
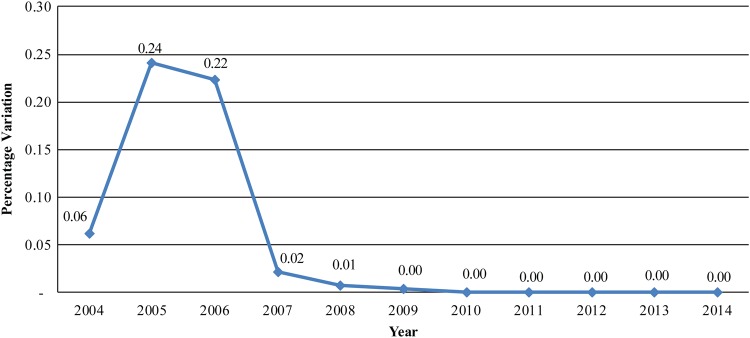
Rate of variation between supplementary immunization activity (SIA) funds disbursed and accounted for, 2004–2014. Data are from Akintola Williams Deloitte financial reports on SIAs prepared for World Health Organization Nigeria.

The rate of financial accountability in the course of reconciling expenditures between the bank's records and the accounting firm's findings has decreased to a negligible margin since 2007 (Figure 5). This was achieved through meticulous tracking of funds and reconciliation processes, which are key to the WHO's stewardship of the resources received from donors and national counterparts.

### Introduction of Mobile Payment Into the DDM in Compliance With National Financial Inclusion Policy

As summarized in Table 2 below, during the first phase of the mobile payment pilot, 97% of the targeted beneficiaries were paid through mobile payment and cashed out at designated agent locations. For those without a mobile phone, arrangements were made to pay them in cash at designated bank branch.

**Table 2.** Number of Vaccination Personnel Paid During the Mobile Payment Project in May 2015

Description	Kaduna	Kano	Overall
Total beneficiaries targeted, no.	967	904	1871
Beneficiaries paid through mobile payment agent, no.	963	847	1810
Beneficiaries paid in cash through the bank, no.	4	57	61
Rate of mobile payment, %	100	94	97

## DISCUSSION

For >11 years, WHO Nigeria's DDM has proved to be an important component of the immunization program in Nigeria by providing an innovative solution toward timely deployment of funds for field activities and direct payment of allowances to thousands of vaccination personnel supporting every round of various SIAs. Detailed financial and management reports are produced in a timely and independent fashion by an accounting firm after thorough reconciliation of payment documents with the disbursing bank. The rate of accountability has improved since 2007, with the variance between bank records and those of the accounting firm decreasing to 0% beginning in 2009.

The systematic engagement of implementing partners and government counterparts throughout the funds management process has contributed immensely to the credibility and transparency of the mechanism [13]. There are a number of checks and balances in the DDM process, with the national authorities playing an active role in selection of vaccination teams, verification of the eligible payees, and certification of vaccination personnel's participation in the campaigns.

The operational challenges the DDM faces mainly arise from the voluminous number and cost of transactions, infrastructural constraints, and security risks. The most cumbersome aspect of the DDM process involves payment of the vaccination personnel. The challenge is compounded by the large number of payees; the poor banking network, especially in the rural part of the country; and the low literacy levels [18]. The WHO regularly convenes consultative meetings with PEI and DDM partners to address these challenges.

One of the steps taken to improve the payment mechanism was the introduction of mobile payments in the DDM. Overall, the first mobile money pilot went well, with 97% of the 1871 targeted beneficiaries receiving their entitlement through the mobile payment scheme. Some of the major challenges encountered in executing the pilot included errors in beneficiary data collection, the poor mobile network, and the poor literacy level of the beneficiaries, which hampered ability to change passwords. The lessons learned from the pilot will be used in scaling up the project. Owing to the limited financial infrastructure in the rural parts of the country, there will still be a significant number of beneficiaries who may not be covered by the new mode of payment. The project should adopt hybrid payment modes to ensure no payee is left out. We anticipate continued reduction of operational cost and better management of financial and security risks once the mobile payment is rolled out over a significant geographic area of the country. While its benefits are appreciated, DDM-implementing partners should also address the customers’ security concerns, as studies show these to be major barriers to the wide use of the mobile payments [19, 20].

The challenges the program faced prior to 2004 were overcome with timely, direct, and full payment of beneficiaries with the successful maintenance of the DDM. Despite prevailing operational and security challenges, the DDM project continues to play an important role in successful implementation of the 2013–2018 polio endgame strategic action plan and the 2011–2020 global vaccine action plan, which require timely deployment of funds at the grassroots level and direct payment to numerous beneficiaries [21, 22].

The DDM was designed to suit the campaign delivery formats of SIAs in Nigeria. With support from WHO Nigeria, Pakistan has successfully replicated the DDM for polio SIAs in that country [23]. As part of polio legacy planning, we recommend adoption of the DDM for other opportunities that involve the engagement of large teams as new vaccines are being introduced in Nigeria and beyond.
